# Laparoscopic patch repair of recurrent anterior diaphragmatic hernia in a child with SMA

**DOI:** 10.1186/2193-1801-3-165

**Published:** 2014-03-31

**Authors:** Noemi Cantone, Francesca Destro, Michele Libri, Stefania Pavia, Mario Lima

**Affiliations:** S. Orsola Malpighi Hospita, Pediatric Surgery Unit, Via Massarenti 11, 40138 Bologna, Italy

## Abstract

An anterior congenital diaphragmatic hernia (CDH) is a diaphragmatic defect that allows the passage of abdominal organs into the thorax. It is typically asymptomatic (the diagnosis is incidental) and it requires surgical correction. In this paper we present a 6 year-old girl affected by spinal muscular atrophy (SMA) who was diagnosed with anterior CDH. Four years after laparoscopic closure of the defect by interrupted suture the girl returned for hernia recurrence. Another laparoscopic procedure was performed and the defect was closed using a GORE-TEX patch. We postulate a mechanism of altered respiratory dynamic and increased abdominal pressure related to scoliosis favouring CDH recurrence in patients with neuromuscular pathologies such as SMA. In these patients patch interposition should be considered in the first place in order to reduce tension over margins. Laparoscopy is a safe and feasible procedure for CDH correction also in case of recurrence and when the interposition of a patch is required.

## Introduction

Anterior congenital diaphragmatic hernia (CDH) consists of a diaphragmatic defect extending from the anterior margin of the central diaphragmatic tendon. Patients are usually asymptomatic or mildly symptomatic because the defect is small. In these cases the diagnosis is incidental (Van De Winkel et al. 2011). Surgical correction is required by primary closure of the defect or through the interposition of a patch (Van De Winkel et al. 2011;Yavuz et al. 2006).

We report a case of laparoscopic correction of recurrent anterior CDH using GORE-TEX ® patch.

## Case report

A 6-year old girl was admitted with suspected CDH. She had type 2 spinal muscular atrophy (SMA) with recurrent respiratory infections. During an episode of respiratory distress, a chest X-Ray showed intestinal gas shadow in the lower thorax and mediastinal shift. A CT scan confirmed bowel loops in the left anterior and lower thorax. Laparoscopic exploration showed a large diaphragmatic defect with the colon protruding through the left chest surrounded by a sac. We reduced the herniated viscera without sac removal and we closed the defect with interrupted non-absorbable suture. There were no complications. The hospital stay lasted 9 days.

**Figure 1:**
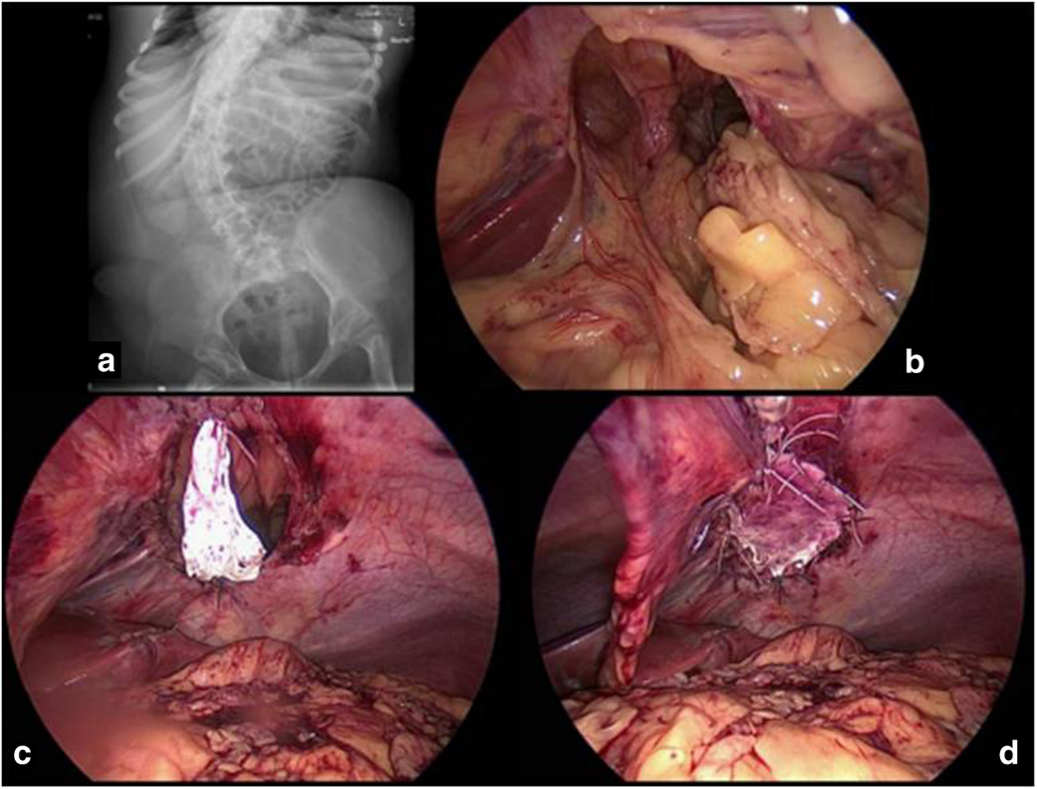
**The x-ray evaluation showed the presence of abdominal organs in the thorax along with severe scoliosis (a); laparoscopic exploration was performed in order to confirm the diagnosis of recurrent congenital diaphragmatic hernia (b) and to close the defect using a prosthetic patch (c, d).**

An asymptomatic recurrence occurred four years later. We performed another laparoscopy that showed the colon and the omentum herniated through a diaphragmatic defect similar to the first one. We used a GORE-TEX ® patch anchored by interrupted suture to close the defect (Figure 1). The girl was discharged after 7 days without any complications. She is now doing well 2 years after surgery.

### Surgical procedure

In both procedures the girl was supine with legs apart and the surgeon standing between them, the assistant on the left side and the scrub nurse on the right one. The first trocar for the 30° optic was inserted through a sopraumbilical incision by open technique. Two additional 5 mm trocars were introduced under direct vision in the right and left flank. A 2/0 Ethibond canoe-shaped needle was used for the synthesis of the defect (with or without patch interposition). Operative times were 2 and 3 hours respectively.

## Discussion

Anterior diaphragmatic hernia is a rare type of hernia that is asymptomatic in most cases. It requires surgical correction that can be achieved by minimally invasive surgery. This kind of surgery is based on the same principles of traditional surgery. In particular laparoscopy has been proved to be safe and effective (Van De Winkel et al. 2011;Marhuenda et al. 2009;Arca et al. 2003;Shah et al. 2009;Mallick & Alqahtani 2009) with faster postoperative recovery (Vijfhuize et al. 2012). Recurrence rate after MIS has been questioned but it seems to be higher only with thoracoscopic surgery. However growing experience is expected to lower the recurrence risk and to shorten the operative time (Vijfhuize et al. 2012).

Regardless of the surgical strategy up to 22% of recurrence rate has been reported (Rowe and Stolar 2003). Identified recurrence risk factors are large defects and specific ventilator strategies (e.g. ECMO) (Hajer et al. 1998). There are still controversies over the use of a patch (Atkinson and Poon 1992;De Kort and Bax 1996) that is required in case of large defects whose closure might be under tension. Tension itself might be a risk factor.

In our case, during the second operation, we decided to use a non-absorbable patch following the principle of a tension-free repair (Dutta and Albanese 2007) and possibly reducing the potential for a future recurrence. This manoeuvre has proved to be easy and with positive results in children (Dutta and Albanese 2007).

We have analysed possible causes of recurrence suggesting the involvement of the underlying disease. In fact SMA is characterized by degeneration of alpha motor neurons of the spinal cord resulting in hypotonia and muscle weakness. The respiratory function is variable compromised, depending on the involvement of intercostal muscles and diaphragm. The latter is usually spared and it acts with frequent, irregular and paradox movements (Polomsky et al. 2009) requiring mechanical ventilation. SMA is also associated with skeletal abnormalities and postural defects as kyphosis and scoliosis (Polomsky et al. 2009). These elements lead to the loss of the diaphragmatic curvature and elasticity. Possible effects are the increment of intra-abdominal pressure (due to the upward displacement of the lower spine) and the risk of hiatal hernia formation, as hypothesized by Bianchi and colleagues (Bianchi et al. 1960).

In the same way they might be risk factors for CDH recurrence although a sure cause and effect relationship is difficult to prove. Our patient had severe scoliosis (Figure 1a), compromised respiratory dynamic and a large diaphragmatic defect, despite the position of the hernia.

The defect closure with laparoscopic patch interposition was effectively completed without difficulties: the abdominal cavity was free from adhesions thanks to the use of laparoscopy also in the first operation.

## Conclusions

Laparoscopic approach is a safe, effective and an aesthetic procedure for CDH repair in children, also in case of recurrence. The use of a patch is suggested in case of large defects or situations that might increase the pressure over the diaphragmatic margins such as neuromuscular pathologies (e.g. SMA).

### Consent

Oral informed consent was obtained from the patient’s guardian for the publication of this report and any accompanying images in an anonymous way.

## Acknowledgement

SP helps collecting information.
